# Ginseng (*Panax quinquefolius*) Reduces Cell Growth, Lipid Acquisition and Increases Adiponectin Expression in 3T3-L1 Cells

**DOI:** 10.1093/ecam/neq051

**Published:** 2011-02-13

**Authors:** Chia-Rou Yeo, Sea-Ming Lee, David G. Popovich

**Affiliations:** Department of Chemistry, National University of Singapore, Science Drive 4, 117543, Singapore

## Abstract

An American ginseng (*Panax quinquefolius*) extract (GE) that contained a quantifiable amount of ginsenosides was investigated for the potential to inhibit proliferation, affect the cell cycle, influence lipid acquisition and adiponectin expression in 3T3-L1 cells. Six fingerprint ginsenosides were quantified by high performance liquid chromatography and the respective molecular weights were confirmed by LC-ESI-MS analysis. The extract contained Rg1 (347.3 ± 99.7 *μ*g g^−1^, dry weight), Re (8280.4 ± 792.3 *μ*g g^−1^), Rb1 (1585.8 ± 86.8 *μ*g g^−1^), Rc (32.9 ± 8 *μ*g g^−1^), Rb2 (62.6 ± 10.6 *μ*g g^−1^) and Rd (90.4 ± 3.2 *μ*g g^−1^). The GE had a dose-dependent effect on 3T3-L1 cell growth, the LC50 value was determined to be 40.3 ± 5 *μ*g ml^−1^. Cell cycle analysis showed modest changes in the cell cycle. No significant changes observed in both G1 and G2/M phases, however there was a significant decrease (*P* < .05) in the S phase after 24 and 48 h treatment. Apoptotic cells were modest but significantly (*P* < .05) increased after 48 h (3.2 ± 1.0%) compared to untreated control cells (1.5 ± 0.1%). Lipid acquisition was significantly reduced (*P* < .05) by 13 and 22% when treated at concentrations of 20.2 and 40.3 *μ*g ml^−1^ compared to untreated control cells. In relation to adiponectin activation, western blot analysis showed that the protein expression was significantly (*P* < .05) increased at concentrations tested. A quantified GE reduced the growth of 3T3-L1 cells, down-regulated the accumulation of lipid and up-regulated the expression of adiponectin in the 3T3-L1 adipocyte cell model.

## 1. Introduction

**Figure 1:**
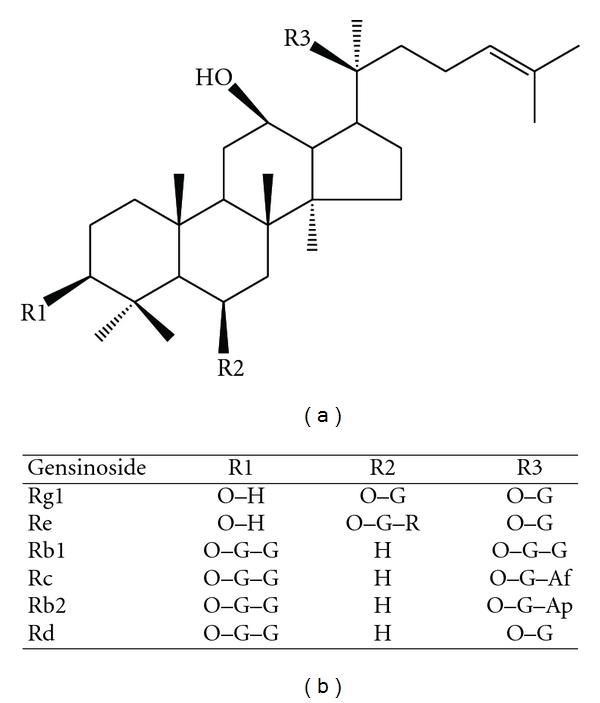
Chemical structure of dammarane-type ginsenoside triterpenoids detected in the GE (*P. quinquefolius*). Regions R1–R3 consist of different attachments of sugar moieties and refer to the following abbreviations: Af: arabinofuranose, Ap: arabinopyranose, G: glucopyranose, R: rhamnopyranose.

There are many natural plant compounds that have been shown to possess bioactive properties. Many of these compounds are plant secondary metabolites that function as deterrents to herbivores [1]. Groups of natural compounds isolated from plants such as polyphenol epigallocatechin-3-gallate (EGCG) from tea (*Camellia sinensis*) [2], vanilloids such as capsaicin from chili peppers (*Capsaicum annuum*) [3] and water extracts from cinnamon (*Cinnamomum zeylanicum*) [4] have all been shown to influence the regulation of cultured adipocytes (3T3-L1 cells). *In vivo*, the molecular regulation of adipocyte is thought to be a contributing factor to the development of the metabolic syndrome and diabetes [5]. These disorders share a common risk factor of obesity. Adipocytes are a metabolically active organ that release a complex set of cytokines that contribute to the maintenance of health or to the development of disease. The increase in adipocyte lipid content can influence adipocyte function such as reducing adiponectin secretion which has been related to insulin resistance and increase risk of diabetes [5, 6]. Reports have shown that ginseng (*Panax quinquefolius*) may improve blood glucose control in normal and diabetic subjects [7]. Animal studies also indicate that ginsenosides, dammarane triterpenoids (Figure 1), such as Re may improve glucose control [8]. It is unclear if an individual ginsenoside or a group of compounds classified into either protopanaxatriol or protopanaxadiol ginsenoside groups [9] such as those contained in an extract or decoction are responsible for these reported effects. The literature on the effect of specific ginsenosides on the promotion or inhibition of adipogenesis is currently unclear. In four reported studies using specific ginsenosides to test the effect on adipogenesis in cultured adipocytes (3T3-L1 cells), two studies reported an inhibition of PPAR*γ*, the master regulator of adipocyte differentiation, using ginsenosides Rh2 and Rg3 [10, 11] and two studies reported increased expression PPAR*γ* and adipogenesis [12, 13]. The objective of this study was to study the effect of a ginseng extract (GE) on adipocyte cell growth, differentiation and lipid acquisition of 3T3-L1 cells and the influence on adiponectin expression. These effects were investigated in terms of its cytotoxic effects on preadipocyte viability, the changes in cell cycle distribution, lipid accumulation after differentiation as well as the expression of adiponectin in the 3T3-L1 cell line, a model system often used to study adipocyte metabolism.

## 2. Methods

### 2.1. Study Design

The effect of American GE (*P. quinquefolius*) with a quantified ginsenoside profile was investigated for a propensity to affect cultured murine fibroblast (3T3-L1) cell growth, cell cycle and to influence lipid acquisition and adiponectin protein expression in the 3T3-L1 cell line.

### 2.2. Extraction of Plant Material

Dried American ginseng (*P. quinquefolius*) was purchased locally, ground and refluxed with methanol (500 ml) three separate times for 3 h each. The extracts were combined, filtered (Whatman no. 4 paper), and the methanol removed under vacuum and re-suspended in distilled water. The extract was then applied to a preconditioned polymeric absorbent Amberlite XAD-4 (Sigma, St. Louis, MO, USA) column (pore diameter of 40 Å, bed volume of 100 cm^3^, average flow rate of 1 ml min^−1^) and was washed with distilled water [11] and ginsenosides were then eluted with absolute ethanol (500 ml) as previously described [14]. The ethanol was removed under vacuum, lyophilized and is herein referred to as the GE.

### 2.3. High Performance Liquid Chromatography and ESI-MS Analysis

To assess the amount of ginsenosides, a high performance liquid chromatography (Waters Alliance 2695, Waters, Milford, MA, USA) was employed with a quaternary gradient pump and a photodiode array (PDA) detector (Waters 2996), controlled by Empower Pro software (Waters). A Waters Symmetry reversed phase C-18 Column (4.6 mm × 250 mm, 5 *μ*m particle size) was used and the mobile phase consisted of distilled water (A) and acetonitrile (B). The column temperature was 25°C, sample injection volume was 20 *μ*L, a flow rate of 1 ml min^−1^, and detection wavelength was set at 203 nm. Two solvent programs were previously described [14], program 1 was used to separate Rb1, Rc, Rb2 and Rd, while program 2 was used to separate Rg1 and Re. Program 1 solvent gradient was as follows at time 0 min, 20% (B), and flow rate of 1 ml min^−1^; 20 min, 25.7% (B), 0.70 ml min^−1^; 40 min, 49% (B), 0.70 ml min^−1^; 50 min, 100% (B), 0.70 ml min^−1^; 60 min, 20% B, 1 ml min^−1^. Program 2 was as follows 0 min, 20% (B), 1 ml min^−1^; 45 min, 22% (B), 0.70 ml min^−1^; 50 min, 60% (B), 0.70 ml min^−1^; 60 min, 20.0% (B), 1 ml min^−1^.

Ginsenoside standards (Rg1, Re, Rb1, Rb2, Rc and Rd) were purchased from Chromadex (Santa Ana, CA, USA) and were used to establish calibration curves. Molecular weight determinations and confirmation of ginsenosides were measured using a Finnigan MAT (San Jose, CA, USA) LCQ quadrupole ion trap MS with MS^n^ capabilities in negative mode. The ESI-MS conditions were set as follow: ion spray voltage of 4.50 kV, capillary voltage of –17 V, capillary temperature of 290°C and were delivered to the MS at a flow rate of 0.4 ml min^−1^. The scanning mass spectra were focused on the *m*/*z* range of 50–1500 U.

### 2.4. Cell Culture

Murine fibroblast cells (3T3-L1) were obtained from ATCC (Manassas, VA, USA). The cells were maintained in Dulbecco's Modified Eagle's media (DMEM) (Sigma) and supplemented with 10% fetal bovine serum (Sigma) and penicillin/streptomycin (100 U ml^−1^) (GIBCO; Invitrogen; Burlington, Canada) in a humidified atmosphere of 5% CO_2_ at 37°C. Cell cultures were maintained at a cell concentration between 2 × 10^3^ and 6 × 10^4^ cells ml^−1^. Cells were subcultured by total media replacement using 0.25% (w/v) trypsin-0.53 mM EDTA solution (GIBCO). Depending on the cell numbers, cells were subcultured in every 2–3 days, and were incubated at 37°C in a 5% CO_2_ humidified incubator. Viable cell numbers were assessed in quadruplicate by using 0.04% trypan blue exclusion dye (MP Biomedicals, OH, USA) via a Neubauer hemocytometer (Blaubrand, Germany). GE was dissolved in DMEM and was passed through a 0.2 *μ*m filter (Millex GP, Ireland) prior to cell treatment.

### 2.5. Cell Viability MTT Assay

Cell growth was assessed using MTT [3-(4,5-dimethylthiazol-2-yl)-2,5-diphenyl tetrazolium bromide] assay to establish an LC50 value (concentration to inhibit 50% of cells). 3T3-L1 cells were seeded at a concentration of 2.5 × 10^4^ cells ml^−1^ in 96-well plates. Controls consisted of cells, culture media without GE. GE was dissolved in culture media and incubated with cells for 72 h. The media was removed and MTT (0.5 mg ml^−1^ dissolved in DMEM) was added and incubated in the dark for 4 h. Acidified sodium dodecylsulfate (SDS) (0.01 M HCL) was added to solubilize the formazan crystal overnight as described previously [15]. The optical density was measured at an absorbance of 570 nm and reference absorbance of 650 nm in a microplate reader (Multiskan Spectrum, Thermo Electro Corporation, Waltham, MA, USA). Cell viability (%) was determined as [mean (absorbance of sample at 570 nm—absorbance of reference sample at 650 nm)/mean absorbance of control] × 100%.

### 2.6. Cell Cycle Analysis

GE was added to 3T3-L1 cells (2.5 × 10^4^ cells ml^−1^) at the LC50 of 40.3 *μ*g ml^−1^ (described subsequently) and incubated at 37°C in a 5% CO_2_ in a humidified incubator for 24, 48 and 72 h. Untreated cells acted as controls at each respective time point. Media was collected and cells were trypsinized, combined with collected media and centrifuged (80 g, 7 min). The supernatant was discarded and cells were washed twice in phosphate buffered saline (PBS). Cells were fixed in ice-cold 70% ethanol and stored overnight at 4°C. Ethanol was removed by centrifugation (500 g, 5 min) and 1 ml of PBS containing 50 *μ*g ml^−1^ propidium iodide (Sigma) and 100 U ml^−1^ RNase A (Applichem Inc., CT, USA) was added and incubated for 30 min in the dark at room temperature. The cell cycle was measured using Guava PCA flow cytometer CytoSoft software (Guava Technologies Inc, Hayward, CA, USA) as described previously [16].

### 2.7. Oil Red O Staining

3T3-L1 cells were seeded in quadruplicate at 2.5 × 10^4^ cells ml^−1^ in six-well plates and were allowed to adhere. Cells were then induced to differentiate utilizing the following schedule of media and hormone additions [0.5 mM 1-isobutyl-3-methylxanthine (IBMX), 1 *μ*M dexamethasone (DEX), 10 *μ*g ml^−1^ insulin]. GE was added during the whole differentiation process in all media preparations and controls consisted of identical media but without the addition of the GE. GE was added at concentrations of 20.2 and 40.3 *μ*g ml^−1^. On Day 2, the media was replaced with initiation media (0.5 mM IBMX, 1 *μ*M DEX). On Day 4, the media was replaced with progression media (10 *μ*g ml^−1^ insulin). On Days 6 and 8, the media was replaced with only GE in media. At Day 10, the media was replaced with 10% formalin in PBS (3 ml) and incubated for 5 min at room temperature. The formalin was replaced by formaldehyde, followed by incubation for 1 h at room temperature and the cells were washed with isopropanol (60%) and allowed to dry. Oil red O working solution was added to each well and left to stand for 10 min as described previously [17]. Oil red O solution was removed and cells were washed with flowing deionized water. Cells were assessed using an Olympus BX51 (U-25ND25-2) microscope with imaging software (Center Valley, PA, USA). To quantify the amount of lipids accumulated, oil red O stain was eluted with 100% isopropanol and absorbance (500 nm) was measured by a spectrophotometer.

### 2.8. Adipocyte Viability and Western Blot Analysis

Differentiated 3T3-L1 cells (described above) were lysed using the cell lytic reagent (Sigma) and centrifuged (13200 g, 15 min). The supernatants were collected for *β*-Actin and adiponectin (Acrp30) western blot analysis. Protein extracts were quantified using BSA method according to manufacturer's protocol (Bio-Rad Laboratories, Hercules, CA, USA) and measured by a spectrophotometer at an absorbance of 595 nm prior to western blot analysis. Polyclonal rabbit anti-adiponectin antibody (Acrp30), polyclonal rabbit *β*-Actin and secondary antibody goat anti-rabbit IgG (H+L) horseradish peroxidase (HRP) were purchased from BST Scientific (Singapore). The protein extract (30 *μ*g per lane) were separated using SDS polyacrylamide gel electrophoresis on 12% polyacrylamide gels (Mini-Protean Tetra Cell, Bio-Rad). Upon separation, proteins were electrophoretically transferred to nitrocellulose (NC) membranes (ClearPAGE, C.B.S. Scientific, Del Mar, CA, USA) by a semi-dry transfer blotter (C.B.S. Scientific). NC membranes were first incubated with 5% skim milk in phosphate-buffered saline with 0.1% Tween-20 (TBST) solution for 1 h to reduce non-specific binding. Acrp30 and *β*-Actin (1 : 1000 dilutions) were then added into separate NC membranes and blotted with 5% skim milk in TBST and were gently agitated overnight at 4°C. NC membranes were incubated with peroxidase-conjugated secondary antibodies (HRP) (1 : 10 000) for 1 h, and washed three times using TBST. *β*-Actin and Acrp30 band intensity were visualized using enhanced chemiluminescence (Thermo Scientific). Protein expressions were visualized using a Fluorchem FC2 Imaging System (Alpha Innotech, San Leandro, CA, USA). Protein expression was quantified densitometrically using the software GelPro 32 (Media Cybernetics, Bethesda, MD, USA).

Guava flow cytometer and ViaCount Reagent (Guava Technologies) were used to assess whether the treatment had an effect on adipocyte viability. 3T3-L1 cells were seeded in six-well plates to a final concentration of 2.5 × 10^4^ cells ml^−1^ and differentiated as described above. Controls consisted of the identical media but without GE and the treatment of cells with GE was at the LC50 concentration of 40.3 ± 5 *μ*g ml^−1^. At Day 10, the media was removed and adherent cells were washed with PBS. Cells were removed by trypsinization (described earlier). Cells were mixed with the Guava ViaCount reagent which can distinguish between viable and non-viable cells based on the differential permeability of two DNA fluorochrome binding dyes, for 5 min in 1.5 ml microcentrifuge tubes. Guava flow cytometry system with CytoSoft software that contains the ViaCount module was used for data acquisition and viable cells were quantified.

### 2.9. Statistical Methods

Data were expressed as mean ± standard deviation (SD) and were analysed using paired *T*-test to compare control with GE. Differences were considered significant compared to control at *P* < .05 . MTT assay was performed with eight replicates in three separate experiments. Both cell cycle analysis and oil red O staining were performed in triplicates in three separate experiments. Western blot analysis was performed with three different cell lysates repeated in three separate experiments on different days.

## 3. Results

### 3.1. Ginsenoside Profile

The GE was assessed for the standard six fingerprint ginsenosides and contained Rg1 (347.3 ± 99.7 *μ*g g^−1^, dry weight), Re (8280.4 ± 792.3 *μ*g g^−1^), Rb1 (1585.8 ± 86.8 *μ*g g^−1^), Rc (32.9 ± 8 *μ*g g^−1^), Rb2 (62.6 ± 10.6 *μ*g g^−1^) and Rd (90.4 ± 3.2 *μ*g g^−1^). The standards and the individual ginsenosides detected in the extract was further assessed using ESI-MS analysis to confirm the molecular weight of the compounds. The molecule ion and fragments are listed in Table 1. All six ginsenosides produced the most abundant ion as the molecule ion [M – H]^−^.

### 3.2. MTT Dose-Response and Cell Cycle Analysis

**Figure 2:**
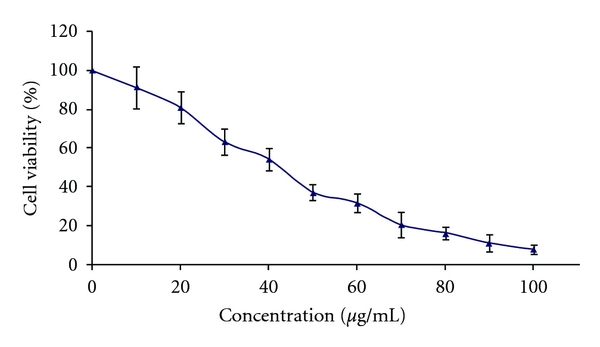
MTT-dose-response relationship of a GE (*P. quinquefolius*) after 72 h incubation with 3T3-L1 cells assessed by an MTT viability assay. Values are expressed as percentage of untreated control cells (mean ± SD).

The MTT dose–response analysis of the effect of GE on the growth of 3T3-L1 cells is shown in Figure 2. The LC50 was calculated from a plot of viability (%) against a log concentration (graph not shown) and yielded a linear equation of *y* = –104.51 × +217.85 (*r* ^2^= 0.9837). The LC50 was calculated to be 40.3 ± 5 *μ*g ml^−1^.

**Figure 3:**
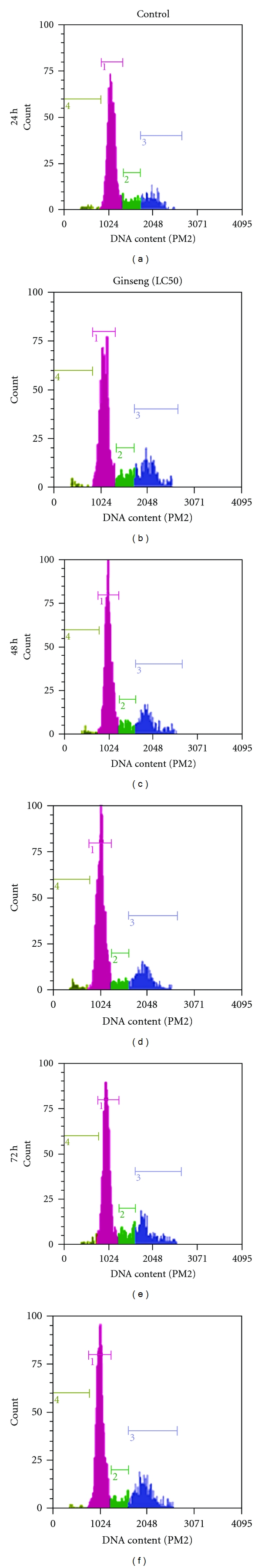
Representative DNA histogram of untreated cells (control) and GE-treated cells for 24, 48 and 72 h. Cells were treated at the LC50 concentration (40.3 *μ*g ml^−1^). DNA histograms shown are representative histograms of three individual experiments, performed in triplicates.

Representative cell cycle histograms of GE-treated cells for 24, 48 and 72 h are shown in Figure 3 and the corresponding cell cycle analysis is listed in Table 2. Generally, GE treatment of 3T3-L1 cells produced modest changes in the cell cycles. The percentages of cells in the S phase were found to be significantly (*P* < .05) decreased after 24 and 48 h of treatment. Sub-G1 analysis also showed a modest but significant increase after 48 h. No significant changes were observed for either G1 or G2/M phases of the cell cycle.

### 3.3. Adipogenesis and Adiponectin Expression

**Figure 4:**
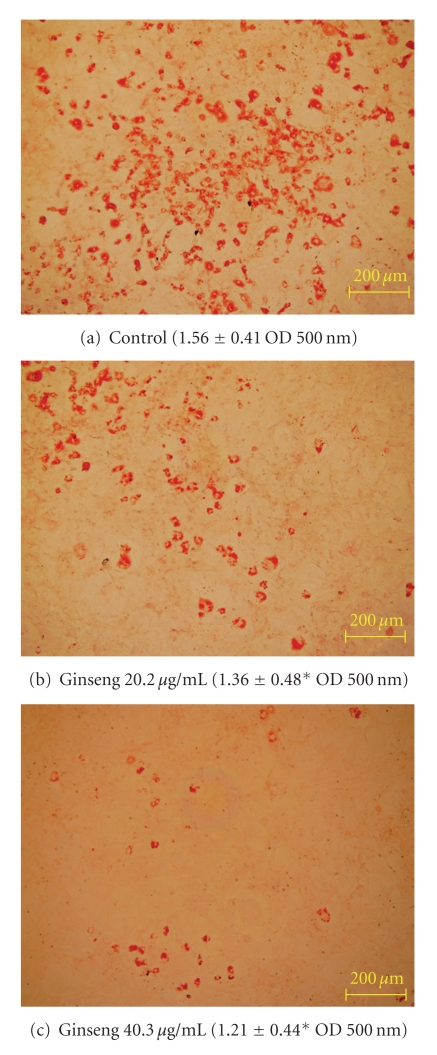
Representative morphological images of lipid uptake in ginseng-treated 3T3-L1 cells and corresponding quantification of lipid stain oil red O in parenthesis. Panels (a)–(c) represent untreated control, ginseng-treated cells at concentrations of 20.2 and 40.3 *μ*g ml^−1^, respectively. Values are expressed as mean ± SD, asterisks indicate a significant difference (*P* < .05) compared to control.

To investigate the effect of GE on 3T3-L1 cell differentiation and corresponding lipid acquisition, cells were differentiated at one half the LC50 (20.2 *μ*g ml^−1^) and the LC50 (40.3 *μ*g ml^−1^) concentrations of the undifferentiated 3T3-L1 cells with untreated cells acting as control. Cells were stained with oil red O and eluted stain quantified (Figure 4). Morphologically, the size of the adipocytes was reduced after both treatments compared to the control. The amount of lipid accumulation by differentiated adipocyte was significantly (*P* < .05) decreased at both concentrations tested. After exposure to a concentration of 20.2 *μ*g ml^−1^, the amount of oil red O lipid stain was significantly reduced (*P* < .05) by 13% compared to the untreated control and decreased by 22% at 40.3 *μ*g ml^−1^.

**Figure 5:**
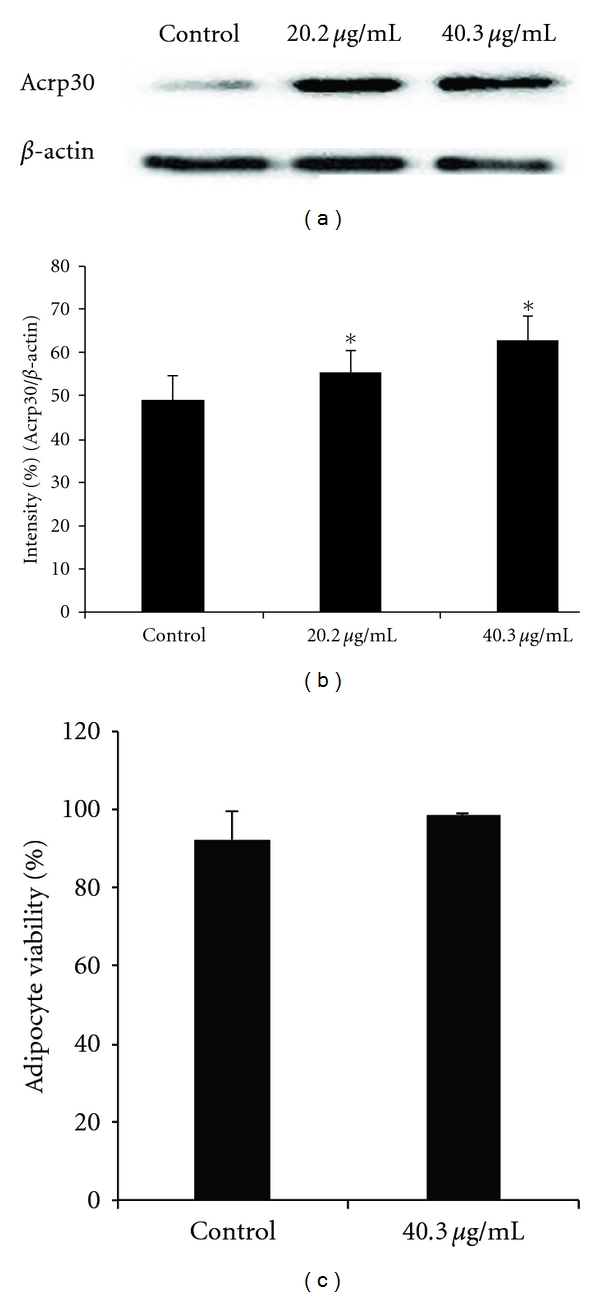
The effect of a GE on the expression of adiponectin (acrp30) in 3T3-L1 adipocytes. Western blot analysis (a) was performed as outlined in ‘Materials and methods' section. Protein expressions were quantified densitometrically and integrated density values were calculated as the density values of the specific protein bands/*β*-actin density values and expressed as a percentage of the control (b) and expressed as mean ± SD of three independent experiments. (c) The viability of the differentiated 3T3-L1 at the concentration used for western blot analysis cell. Asterisks indicate a significant different (*P* < .05) compared to control (Con).

**Figure 6:**
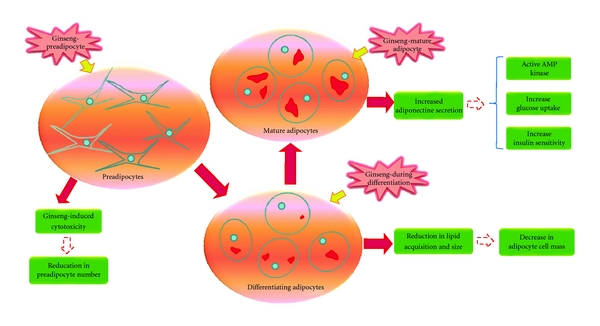
Schematic representation of the preadipocytes, differentiating and mature adipocytes used in this study and result obtained (red arrows). The possible implications are represented by red dotted arrows. Solid red color inside the cells represents Oil Red O stained lipids and small blue circle represents the nucleus.

Figure 5(a) shows the representative western blots of 3T3-L1 cells which were induced to differentiate after being treated with at 20.2 and 40.3 *μ*g ml^−1^, with non-treated as control. Adiponectin expression significantly (*P* < .05) increased at both of the concentrations tested compared to the control. The band intensities were calculated as the density values of the adiponectin protein bands/*β*-actin density values and expressed as a percentage of *β*-actin (Figure 5(b)). It is also noteworthy that after differentiation GE added at the undifferentiated LC50 value of 40.3 *μ*g ml^−1^ did not have a significant (*P* < .05) effect on adipocyte viability as measured by the ViaCount dual DNA fluorochrome binding dyes (Figure 5(c)) which can distinguish between viable and non viable cells. A schematic representation of the results obtained and potential implications are outlined in Figure 6.

## 4. Discussion

In this study, we have shown that a GE that contained a quantified amount of fingerprint ginsenosides had a dose-dependent effect on 3T3-L1 cell growth. Protopanaxatriol type ginsenoside Re, having two glycoside attachments (C6 and C22 of the ring structure) was found to be the most abundant in the extract. Re is typically the most abundant ginsenosides reported in American ginseng (*P. quinquefolius)* [18]. Ginsenosides Rb1, having two glycosides moieties attached at positions C3 and C22 is the most abundance ginsenosides in Asian ginseng (*P. ginseng)* and has been reported to inhibit proliferation of 3T3-L1 preadipocytes cells in a dose dependent manner [13]. Minor ginsenosides Rg3, Rh2 and Rk1 which are not typically found in methanolic extracts [18] have also been shown to have an effect on 3T3-L1 cell growth [10, 11, 19]. The effect of GEs [14, 20], and individual ginsenosides [9, 21] on a variety cultured cancer cells has been well documented and have shown to reduce cell growth while inducing apoptotic death. A reduction in viable cancer cells have been reported according to the hydrophobicity of the ginsenosides with cytoxicity generally increasing as polarity decreases [9]. An interaction with membrane cholesterol has been suggested as a plausible site of action of triterpenoids [1, 22] thereby causing membrane permeation [9] and apoptosis. In this study, 3T3-L1 cells which are murine fibroblast cells did not undergo apoptosis to a significant extent with only a small 1.7% increase in sub-G1 cells noted after 48 h and influenced only other minor changes on the progression of the cell cycle. However, a reduction in cell growth was observed. A reduction in the preadipocyte cell number may lead to an overall reduction in adipocyte tissue mass through adipocyte hyperplasia [23]. Preadipocytes are fibroblasts that are undifferentiated adipocytes and by reducing preadipocyte cell number or preventing differentiation may affect overall lipid acquisition [23] and potential disease risk.

3T3-L1 cells undergo cellular differentiation from a preadipocyte to an adipocyte-like cell when exposed to a hormonal cocktail of insulin, dexamethasone and IBMX [24, 25]. During the course of the 10-day differentiation period, treatment with GE significantly reduced the lipid adipocyte accumulation measured by oil red O lipid staining. The aglycone of ginsenoside Re, protopanaxatriol has been reported to increase lipid uptake and increase PPAR*γ* protein expression which is a regulator of adipocyte differentiation. Conversely, ginsenoside Rh2, a protopanaxadiol-type ginsenoside inhibited adipogenesis by activating AMP kinase (5′-AMP-activated protein kinase) which is a key enzyme involved in lipid and cellular energy regulation [10]. AMP kinase promotes glucose uptake into skeletal muscle and reduces hepatic glucose output non-insulin-dependently [26]. Ginsenoside Rg3 was also reported to inhibit PPAR*γ* and activate AMP kinase [11] and attenuated the effect of rosiglitazone, a known PPAR*γ* ligand. GE used in this study had 38.3 ± 3.7% of the extract as ginsenoside Re and ginsenoside Re has been shown to suppress NF-*κ*B activation and increase glucose uptake in 3T3-L1 cells [27]. Adiponectin expression was enhanced after GE treatment. Adiponectin is the major adipokine secreted by adipocytes and is recognized as a key regulator of insulin sensitivity. Circulating levels of adiponectin are inversely correlated to body mass index [28] and are reduced in obese and insulin-resistant states [24, 29, 30]. Adiponectin has been shown to activate AMP kinase and glucose utilization in mice and muscle cells [31]. *In vivo,* GEs have improved glucose tolerance in diabetic patients [7], but it is unclear as to the molecular mechanism, a plausible target would be adiponectin expression. There is a need for further research both at the cellular and clinical levels to provide additional mechanistic evidence of the interactions between ginsenosides and adipokine signaling.

We have shown that an American GE, containing Re as the most abundant ginsenoside, reduced preadipocyte cell growth, lipid accumulation and increased the expression of adiponectin in the 3T3-L1 cell line. Further experiments are needed to associate these effects with specific ginsenosides or combinations of these contained in extracts and to elucidate a specific molecular mechanism.

## Funding

Financial support and a graduate scholarship from Ministry of Education, Singapore and National University of Singapore (NUS) (Grants R-143-050-287-133/101) (to C.R.Y.).

## Figures and Tables

**Table 1:** ESI-MS ion fragments of the six major ginsenosides.

Ginsenoside	Empirical	Molecular	Ginsenoside content	Main ion fragments, *m/z*	
retention order	formula	weight (Da)	within extract (*μ*g g^−1^)	[M – H]^−^	Others
Rg1	C_42_H_72_O_14_	801.01	347.3 ± 99.7	799.4	859.3, 945.4
Re	C_48_H_82_O_18_	947.15	8280.4 ± 792.3	945.7	1005.2, 799.6
Rb1	C_54_H_92_O_23_	1109.29	1585.8 ± 86.8	1107.6	1031.4, 1163.2, 1193.1
Rc	C_53_H_90_O_22_	1079.27	32.9 ± 8.0	1077.5	329.5, 1031.3, 1149.5
Rb2	C_53_H_90_O_22_	1079.27	62.6 ± 10.6	1077.5	329.5, 945.5, 1005.2, 1149.5
Rd	C_48_H_82_O_18_	947.15	90.4 ± 3.2	945.6	1005.2, 849.7, 1059.2

**Table 2:** Cell cycle distribution of 3T3-L1 cells treated with GE.

Phase in cell cycle	Incubation period (h)	Control^(a)^ (%)	GE^(b)^ (%)	*P* -value
Sub G1	24	1.2 ± 0.5	2.8 ± 0.5	.20
	48	1.5 ± 0.1	3.2 ± 1.0	.04*
	72	1.3 ± 0.4	1.3 ± 0.8	.36

G0/G1	24	58.4 ± 5.5	58.0 ± 6.7	.73
	48	65.7 ± 3.8	62.1 ± 4.1	.88
	72	68.1 ± 6.3	69.7 ± 4.0	.11

S	24	11.8 ± 2.4	9.5 ± 2.2	.03*
	48	8.0 ± 1.7	7.0 ± 1.2	.03*
	72	7.0 ± 3.0	5.6 ± 1.5	.40

G2/M	24	28.7 ± 6.2	30.1 ± 4.6	.95
	48	24.9 ± 2.0	27.7 ± 3.7	.53
	72	23.6 ± 4.6	23.2 ± 3.0	.10

^(a)^Control cells consisted of untreated cells.

^(b)^Tested at LC50 concentration (40.3 *μ*g ml^−1^).

*Significant difference from the corresponding control value.

## References

[1] Wink M (2008). Evolutionary advantage and molecular modes of action of multi-component mixtures used in phytomedicine. *Current Drug Metabolism*.

[2] Lee M-S, Kim C-T, Kim I-H, Kim Y (2009). Inhibitory effects of green tea catechin on the lipid accumulation in 3T3-l1 adipocytes. *Phytotherapy Research*.

[3] Hsu C-L, Yen G-C (2007). Effects of capsaicin on induction of apoptosis and inhibition of adipogenesis in 3T3-L1 cells. *Journal of Agricultural and Food Chemistry*.

[4] Roffey B, Atwal A, Kubow S (2006). Cinnamon water extracts increase glucose uptake but inhibit adiponectin secretion in 3T3-L1 adipose cells. *Molecular Nutrition and Food Research*.

[5] Gil-Campos M, Cañete R, Gil A (2004). Adiponectin, the missing link in insulin resistance and obesity. *Clinical Nutrition*.

[6] Gregoire FM (2001). Adipocyte differentiation: from fibroblast to endocrine cell. *Experimental Biology and Medicine*.

[7] Vuksan V, Sievenpiper JL, Koo VYY (2000). American ginseng (*Panax quinquefolius* L) reduces postprandial glycemia in nondiabetic subjects and subjects with type 2 diabetes mellitus. *Archives of Internal Medicine*.

[8] Xie J-T, Mehendale SR, Li X (2005). Anti-diabetic effect of ginsenoside Re in ob/ob mice. *Biochimica et Biophysica Acta*.

[9] Popovich DG, Kitts DD (2002). Structure-function relationship exists for ginsenosides in reducing cell proliferation and inducing apoptosis in the human leukemia (THP-1) cell line. *Archives of Biochemistry and Biophysics*.

[10] Hwang J-T, Kim S-H, Lee M-S (2007). Anti-obesity effects of ginsenoside Rh2 are associated with the activation of AMPK signaling pathway in 3T3-L1 adipocyte. *Biochemical and Biophysical Research Communications*.

[11] Hwang J-T, Lee M-S, Kim H-J (2009). Antiobesity effect of ginsenoside Rg3 involves the AMPK and PPAR-*γ* signal pathways. *Phytotherapy Research*.

[12] Han KL, Jung MH, Sohn JH, Hwang JK (2006). Ginsenoside 20S-protopanaxatriol (PPT) activates peroxisome proliferator-activated receptor gamma (PPARgamma) in 3T3-L1 adipocytes. *Biological & Pharmaceutical Bulletin*.

[13] Shang W, Yang Y, Jiang B (2007). Ginsenoside Rb1 promotes adipogenesis in 3T3-L1 cells by enhancing PPAR*γ*2 and C/EBP*α* gene expression. *Life Sciences*.

[14] Popovich DG, Yeo SY, Zhang W Ginseng (*Panax quinquefolius*) and Licorice (*Glycyrrhiza uralensis*) root extract combinations increase hepatocarcinoma cell (Hep-G2) viability.

[15] Zhang W, Popovich DG (2008). Effect of soyasapogenol A and soyasapogenol B concentrated extracts on Hep-G2 cell proliferation and apoptosis. *Journal of Agricultural and Food Chemistry*.

[16] Zhang W, Yeo MC, Tang FY, Popovich DG (2009). Bioactive responses of Hep-G2 cells to soyasaponin extracts differs with respect to extraction conditions. *Food and Chemical Toxicology*.

[17] Janderová L, McNeil M, Murrell AN, Mynatt RL, Smith SR (2003). Human mesenchymal stem cells as an *in vitro* model for human adipogenesis. *Obesity Research*.

[18] Popovich DG, Kitts DD (2004). Generation of ginsenosides Rg3 and Rh2 from North American ginseng. *Phytochemistry*.

[19] Chu C-Y, Lee H-J, Chu C-Y, Yin Y-F, Tseng T-H (2009). Protective effects of leaf extract of Zanthoxylum ailanthoides on oxidation of low-density lipoprotein and accumulation of lipid in differentiated THP-1 cells. *Food and Chemical Toxicology*.

[20] Popovich DG, Kitts DD (2004). Mechanistic studies on protopanaxadiol, Rh2, and ginseng (*Panax quinquefolius*) extract induced cytotoxicity in intestinal Caco-2 cells. *Journal of Biochemical and Molecular Toxicology*.

[21] Kitts DD, Popovich DG, Hu C (2007). Characterizing the mechanism for ginsenoside-induced cytotoxicity in cultured leukemia (THP-1) cells. *Canadian Journal of Physiology and Pharmacology*.

[22] Popov AM (2002). A comparative study of the hemolytic and cytotoxic activities of triterpenoids isolated from ginseng and sea cucumbers. *Izvestiya Rossiiskoi Akademii Nauk—Seriya Biologicheskaya*.

[23] Camp HS, Ren D, Leff T (2002). Adipogenesis and fat-cell function in obesity and diabetes. *Trends in Molecular Medicine*.

[24] Abbasi F, Chu JW, Lamendola C (2004). Discrimination between obesity and insulin resistance in the relationship with adiponectin. *Diabetes*.

[25] Rudich A, Tlrosh A, Potashnik R, Hemi R, Kanety H, Bashan N (1998). Prolonged oxidative stress impairs insulin-induced GLUT4 translocation in 3T3-L1 adipocytes. *Diabetes*.

[26] Hegarty BD, Turner N, Cooney GJ, Kraegen EW (2009). Insulin resistance and fuel homeostasis: the role of AMP-activated protein kinase. *Acta Physiologica*.

[27] Zhang Z, Li X, Lv W (2008). Ginsenoside Re reduces insulin resistance through inhibition of c-Jun NH2-terminal kinase and nuclear factor-*κ*B. *Molecular Endocrinology*.

[28] Arita Y, Kihara S, Ouchi N (1999). Paradoxical decrease of an adipose-specific protein, adiponectin, in obesity. *Biochemical and Biophysical Research Communications*.

[29] Tschritter O, Fritsche A, Thamer C (2003). Plasma adiponectin concentrations predict insulin sensitivity of both glucose and lipid metabolism. *Diabetes*.

[30] Weyer C, Funahashi T, Tanaka S (2001). Hypoadiponectinemia in obesity and type 2 diabetes: close association with insulin resistance and hyperinsulinemia. *Journal of Clinical Endocrinology and Metabolism*.

[31] Yamauchi T, Kamon J, Minokoshi Y (2002). Adiponectin stimulates glucose utilization and fatty-acid oxidation by activating AMP-activated protein kinase. *Nature Medicine*.

